# Chronologic Presentation of a Severe Case of Progressive Hemifacial Atrophy (Parry-Romberg Syndrome) with the Loss of an Eye

**DOI:** 10.1155/2014/703017

**Published:** 2014-11-18

**Authors:** Mesut Kaya, Ceyda Sel Yilmaz, Hanifi Kurtaran, Mehmet Gunduz

**Affiliations:** Department of Otolaryngology Head & Neck Surgery, Turgut Özal University Hospital, Faculty of Medicine, Turgut Özal University, Alparslan Turkes Caddesi No. 57, 06510 Ankara, Turkey

## Abstract

Progressive hemifacial atrophy, also known as Parry-Romberg syndrome, is a slowly advancing degenerative disease that mostly affects the cutaneous, subcutaneous fatty tissue, muscle tissue, and bone structures on one side of the face. We describe the chronological progression of this very rare syndrome from early childhood until adulthood in a patient who developed severe atrophy and lost one eye. We also discuss the aetiology and pathophysiology of this syndrome.

## 1. Introduction

Parry, in 1825, and Romberg, in 1846, described cases of progressive hemifacial atrophy (PHA), which is also known as Parry-Romberg syndrome [1, 2]. This syndrome generally affects unilateral cutaneous, subcutaneous fatty tissue, muscle tissue, and rarely bone structures, linked to the dermatomes of the fifth cranial nerve. The syndrome occasionally affects the upper or lower extremities, or the entire body [3]. PHA is a slowly progressive syndrome that usually emerges between 1st and 2nd decades. Atrophy generally progresses for 2–20 years and then stabilises [3]. PHA is more common in females than in males and rarely shows a familial history [4].

Although some authors have suggested that PHA is a variant of scleroderma, others have suggested that it is a different disease entity. Nevertheless, the exact aetiology and pathogenesis of this rare syndrome are unknown. Trauma, encephalitis, immunologic anomalies, cranial vascular anomalies, hyperactivity of the sympathetic nervous system, and slow viral infections were proposed to contribute to the aetiology of PHA [3, 5–7].

The objective of therapy for PHA is to stop its progression in early phases and then ameliorate its symptoms. Immunosuppressive therapy is feasible in patients with cerebral involvement. Once the progression of PHA has stabilised, the atrophic side of the face can be surgically reconstructed [7].

## 2. Case Report

**Figure 1:**
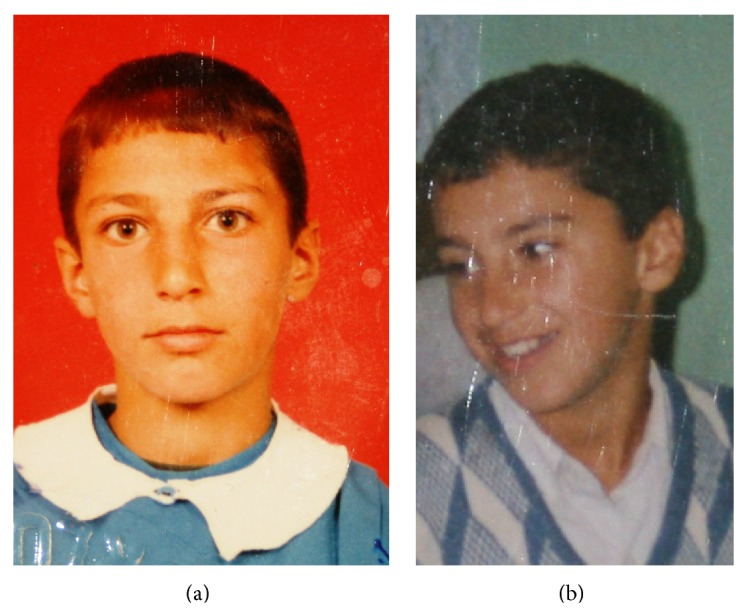
(a) At 8 years of age, very slight atrophy of the left side of the patient's face was apparent. (b) The patient at 10 years of age.

A 23-year-old male patient was referred to our tertiary academic centre because of a defect affecting one side of his face. The patient underwent adenotonsillectomy at 6 years of age, and numbness started to affect the left side of his face 3 months after surgery. At that time, he was informed by his physician, who performed the operation, that there were no problems. However, the numbness affecting the left side of his face increased, and shrinkage and weakness of the left side of the face occurred over the next 2 years. In ten years after surgery, his complaints and symptoms increased (Figures 1(a) and 1(b)) but he could not see the doctor for socioeconomic reasons.

**Figure 2:**
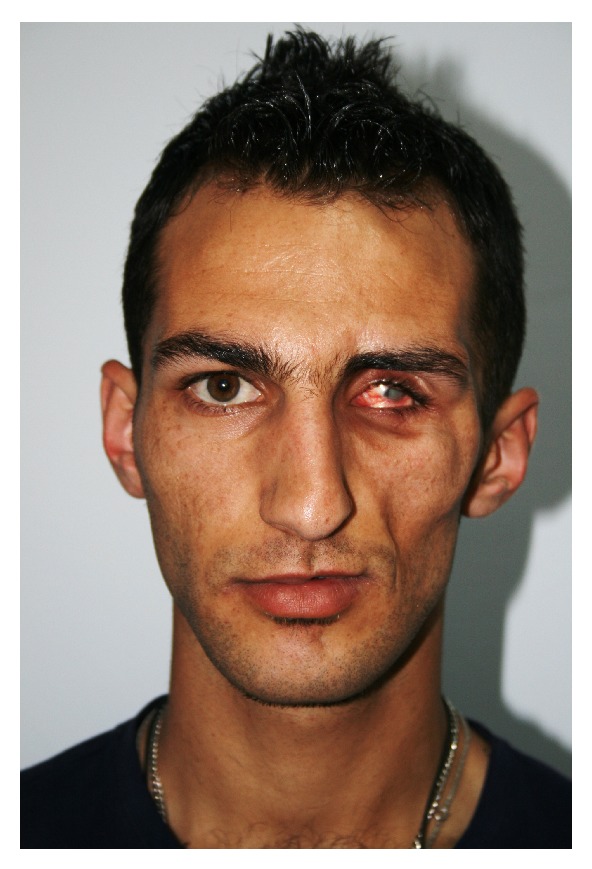
Frontal view of the patient at 23 years of age, showing severe atrophy of fat and muscle tissue, and of the zygomatic arch on the left side, as well as shrinkage of the left eyeball and corneal atrophy.

**Figure 3:**
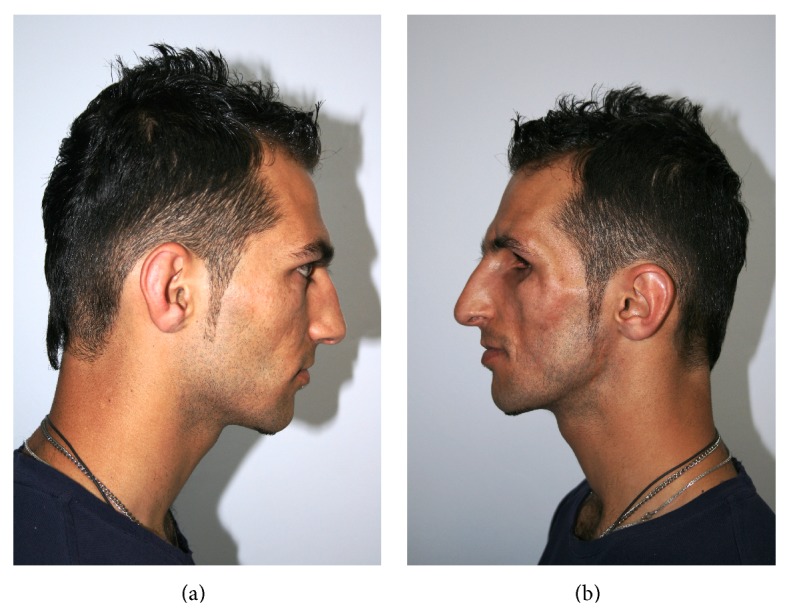
(a) Right view. (b) Left view.

**Figure 4:**
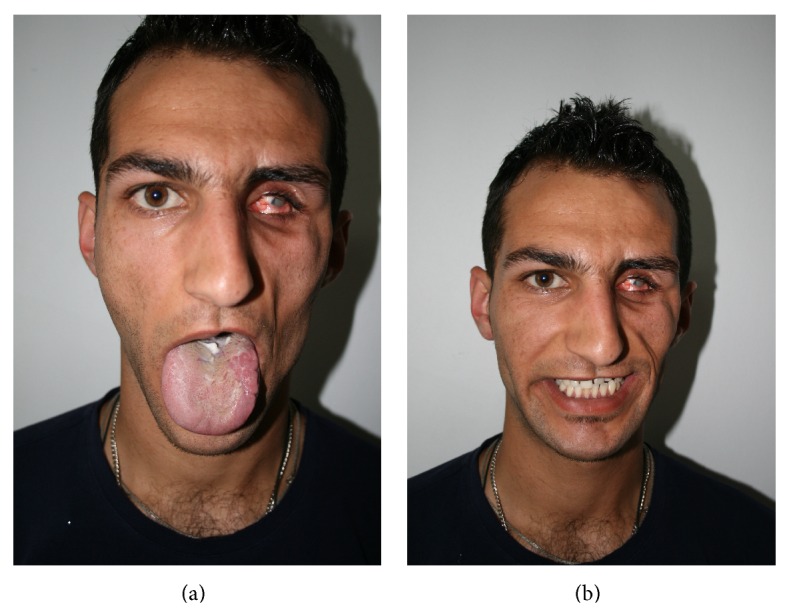
(a) Atrophy and fissuring of the left hemiglossus. (b) Maxillary atrophy has displaced the left upper teeth in an upward and backward direction.

A physical examination at the time of referral to our centre revealed atrophy of cutaneous and subcutaneous fat tissue on the left side of the patient's face, phthisis of the left ocular bulb and corneal atrophy, left zygomatic atrophy, and asymmetry (Figures 2, 3(a), and 3(b)). Hemiglossal atrophy and an increased size of fissures of the left side of the tongue were also seen (Figure 4(a)). Maxillary atrophy displaced the left upper teeth in an upward and backward direction (Figure 4(b)).

**Figure 5:**
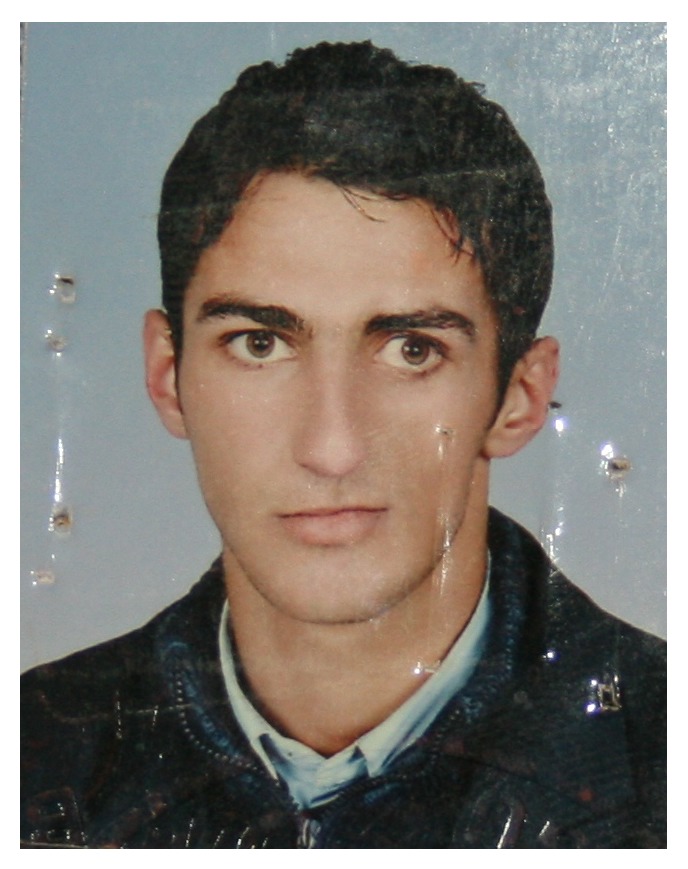
The patient at 16 years of age, 1 year after the first autologous fat injection.

**Figure 6:**
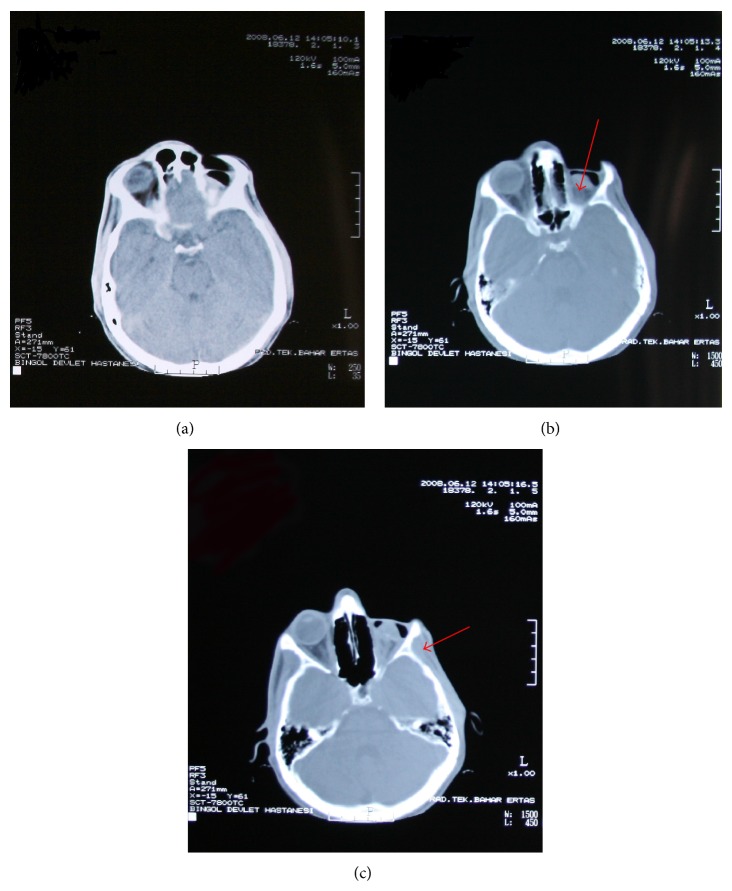
Axial computed tomography images of the head. (a) The bilateral hemispheres are normal in appearance. (b) Atrophy of the left retrobulbar fat tissue and phthisis of the eyeball (arrow) are evident. (c) The left temporal muscle (arrow) is atrophic.

**Figure 7:**
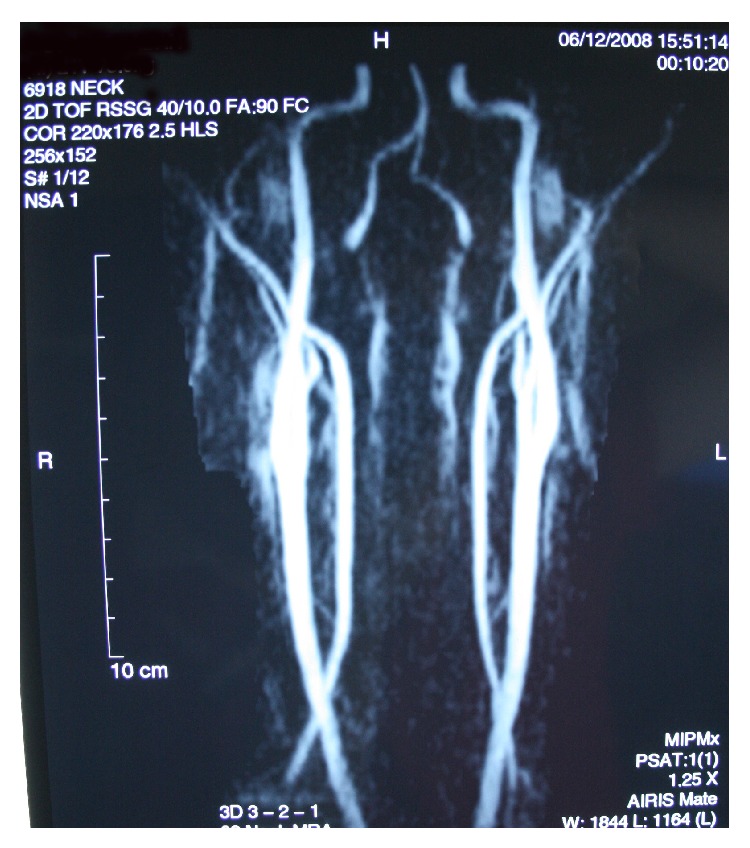
Magnetic resonance angiography showing normal carotid vascularity.

The patient had no dermal lesions or involvement of the extremities. There was no family history of PHA. The patient underwent autologous fat injection at 14 and 16 years of age, but the desired result was not sustained and the atrophy continued (Figure 5). No antibodies were detected in serological tests. Sedimentation and core reactive protein levels were within normal limits, suggesting that the facial disorders were not caused by inflammatory diseases. Computed tomography revealed no apparent neurodegeneration (Figure 6(a)), eliminating Rasmussen encephalitis (RE) from the differential diagnosis. The computed tomographic images revealed a decrease in left retroorbital fat tissue and atrophy of the ocular bulb (Figure 6(b)). The left temporal muscle was atrophic compared with the right temporal muscle (Figure 6(c)). Magnetic resonance angiography was performed to detect possible vascular causes but revealed no vascular abnormalities of the internal and external carotid arteries or of their branches (Figure 7).

The patient was informed that surgical reconstruction of his facial asymmetry would not stop the progression of the disorder and that the cosmetic results would probably be temporary. The patient did not wish to undergo any surgical interventions.

## 3. Discussion

PHA, also known as Parry-Romberg syndrome, is a rare disorder with a largely unknown aetiology that is usually associated with atrophy of cutaneous, subcutaneous fatty tissue, muscle tissue, and rarely bone structures, on one side of the face [3].

Although many theories have been proposed to explain the aetiology and pathogenesis of PHA, the exact cause is still unknown. Heredity, autoimmune disorders, trauma, hypo- or hyperreactivity of the sympathetic nervous system, trigeminal nerve disorders, and infectious diseases have been suggested to play a role in the aetiology of PHA [3]. It was also suggested that trauma plays a pivotal role because 24%–34% of patients with PHA had a history of trauma [7]. Surgical traumas may include thyroidectomy and dental extraction [8]. Obstetric trauma may also play a role in the aetiology of PHA [9]. The family history of PHA in some cases suggests that there is genetic predisposition for this syndrome in some patients [7, 10].

The autoimmune theory of PHA relies on the cooccurrence of PHA with autoimmune diseases [7, 9]. The detection of anti-double-stranded DNA, anti-centromere, anti-cardiolipin, and anti-histone antibodies, as well as rheumatoid factor and an oligoclonal band in cerebrospinal fluid, supports this hypothesis [7, 11]. Slow viral or bacterial infections might also cause PHA, although no organisms were detected in cerebrospinal fluid in prior cases [7, 9].

Cory et al. suggested that PHA is caused by sympathetic nerve hyperactivity, especially superior cervical ganglion inflammation [6]. In an animal study, Resende et al. reported that ablation of the superior cervical ganglion caused hemifacial atrophy, localised alopecia, corneal ulceration, keratitis, strabismus, enophthalmos, ocular atrophy, and slight bone atrophy [12]. These results are supported by those reported by Moss and Crikelair, who performed cervical sympathectomy in rats [13].

PHA often coexists with other neurological, cardiac, ophthalmological, rheumatological, maxillofacial, and orthodontal disorders. Fifteen percent of patients were reported to have neurological disorders, prompting some authors to refer to PHA as a neurocutaneous syndrome [14]. The most common concomitant disorders include migraine, hemiplegia, brain atrophy, and intracranial vascular anomalies, while other symptoms include enophthalmos, uveitis, retinal vasculitis, eyelid atrophy and ptosis, and dental abnormalities. Ruhin et al. hypothesised that dental abnormalities could help to determine the age at the start of PHA [9].

Frontal linear scleroderma, also known as* en coup de sabre*, is a localised form of scleroderma that is usually the initial sign of PHA. The starting age, disease progression, courses of neurological and ophthalmological findings, and the need for immunosuppressive therapy are similar in both frontal linear scleroderma and PHA [7]. Although some authors advocate that frontal linear scleroderma and PHA are not the same entities, others have proposed that PHA is a form of scleroderma [7, 15].

RE is another disease that should be considered in the differential diagnosis of cases like ours. RE is a degenerative disease that advances with progressive cortical inflammation and unilateral hemispheric destruction [16]. It was suggested that PHA and RE are caused by common autoimmune factors and have common antibodies [16]. Similar neurological findings, unilateral involvement, and early onset are common features of RE, and they should be considered in the differential diagnosis [7].

Guerrerosantos et al. divided patients into four groups according to the extent of tissue atrophy before deciding on the therapy [17]. In their classification, type 1 patients had a weakness and slight depression of their facial structures, corresponding to the acute phase of the disease [17]. Mild physical deformities can be noticed by the patient's family or close acquaintances. Type 2 patients have a greater weakness without bone or cartilage involvement. The deformities are more apparent than in type 1 patients and can be noticed by anyone [17]. Type 3 patients have greater weakness of soft tissue and of bone and cartilage structures. The deformities are significant [17]. In patients with type 4 PHA, the atrophy is very severe, and the skin is almost attached to the bone. The advanced bone and cartilage involvement is greater than that in type 3. Patients with type 4 PHA also show functional problems of their lips and nose [17].

Fat and cartilage grafts, silicone injections, prosthesis, bovine collagen injection, and inorganic implants have been used to correct the atrophic facial appearance [18, 19]. Guerrerosantos et al. suggested that their classification could be used to select the appropriate therapy [17]. For type 1 and 2 patients, they reported successful outcomes of fat injection and grafting into the muscle and periosteum or under the submucosal aponeurotic system because of the good vascularisation [17]. Meanwhile, fat injection aimed at feeding the tissue and reconstructing the facial contour may be required in patients with type 3 or 4 PHA. To achieve this, Guerrerosantos et al. suggested that a galeal flap should be used instead of dermal, fat, or muscle tissue flaps, to provide greater volume and tissue softness [17, 20].

In our case, in which PHA was probably caused by adenotonsillectomy, autologous fat injection was performed twice, but these injections did not stop the progression and the patient ultimately developed severe atrophy. Unlike earlier cases, the chronological progression of PHA from childhood to adulthood was documented using photographs. Our literature review revealed that most of the cases had mild or moderate PHA, and its progression stopped before the loss of eyeball. Our case had type 4 PHA, which is the most severe form. Despite performing an extensive battery of serologic tests, we found no abnormalities to explain the cause of this syndrome.
